# Prognostic value of lymphovascular space invasion in stage IA to IIB cervical cancer: A meta-analysis

**DOI:** 10.1097/MD.0000000000033547

**Published:** 2023-04-14

**Authors:** Yuan Huang, Weibo Wen, Xiangdan Li, Dongyuan Xu, Lan Liu

**Affiliations:** a Department of Pathology, Yanbian University Hospital, Yanji, China; b Department of Nuclear Medicine, Yanbian University Hospital, Yanji, China; c Center of Morphological Experiment, Medical College of Yanbian University, Yanji, China.

**Keywords:** cervical cancer, lymphovascular space invasion, meta-analysis, prognosis

## Abstract

Lymphovascular space invasion (LVSI) is a prognostic factor in the existing TNM classification system. The present meta-analysis assessed the role of LVSI in predicting the prognosis of stage IA to IIB cervical cancer (CC).

**Materials and methods:**

PubMed, EMBASE, and Cochrane Library electronic databases were searched to determine relevant articles published in the English language. Our search deadline was May 2022. Critical Appraisal of Prognostic Studies was used to assess the quality for each article. Pooled hazard ratios (HRs) were used to evaluate the performance of LVSI in prognosis prediction.

**Results:**

We enrolled 8 studies involving 25,352 patients published after 2010. Thus, high LVSI was an unfavorable factor in predicting overall survival (HR, 2.08; 95% confidence interval, 1.63–2.66; *P* = .006) and disease-free survival (HR, 2.20; 95% confidence interval, 1.79–2.70; *P *= .000) for patients with CC. However, the disease-free survival and overall survival were significantly different on univariate analysis based on the subgroup analysis stratified by analysis method, but no obvious heterogeneity was found across diverse articles.

**Conclusions:**

The present study showed that LVSI predicts the poor prognostic outcome of stage IA to IIB CC. However, well-designed clinical articles should further assess the independent prognosis prediction performance of LVSI in CC.

## 1. Introduction

Cervical cancer (CC) is the fourth most common cause of morbidity and mortality among malignant tumors in women. In 2020, 604,000 patients were newly diagnosed with CC, with 342,000 deaths reported worldwide.^[1]^ Surgery is the most favorable modality to treat stages ≤ IIA CC classified based on the International Federation of Gynecology and Obstetrics, whereas chemoradiotherapy has been recommended for more advanced stages.^[2,3]^ The elevated lymph node dissection level affects the metastasis number based on normalized pN classification. Moreover, it possibly influences the altered tumor TNM stage while affecting prognosis prediction performance.^[4,5]^ An important factor affecting CC prognostic outcome is lymph node metastasis (LNM).^[6,7]^ In addition, myometrial invasion is considered the first well-recognized sign of aggressive behavior, whereas positive lymphovascular space invasion (LVSI) may predict an increased LNM risk, indicating an increased relapse risk, and it may be the factor that can independently predict prognosis.^[8–10]^

The survival prediction of stage IA to IIB CC based on LVSI is still controversial. Some studies reported that increased LVSI was associated with the poor prognostic outcome of CC.^[11,12]^ However, the association was not detected in the study by Haesen et al.^[13]^ Consequently, our study was conducted to analyze the prognosis prediction performance of LVSI for patients with stage IA to IIB CC.

## 2. Material and methods

### 2.1. Registration

This study was reported following the guidelines of the preferred reporting items of the systematic review and meta-analysis.^[14]^ Ethical approval or patient consent was not necessary because of our study’s retrospective nature.

### 2.2. Study search and selection procedure

#### 2.2.1. Search strategy.

We comprehensively searched Embase, PubMed, and Cochrane Library databases using keywords such as “lymphovascular space invasion” AND “cervical cancer” OR “cervical neoplasm.” Our search deadline was May 2022. The search procedure was implemented until no new relevant articles were detected. Moreover, the reference lists of the articles were also examined to determine potential studies. Eventually, 2 authors evaluated articles in line with the pre-set criteria.

#### 2.2.2. Study screening.

First, the articles were searched using keywords, and irrelevant articles were removed after evaluating their titles and abstracts. Second, the rest of the articles were screened according to study eligibility standards. We included articles conforming to the following criteria: patients pathologically diagnosed with stage IA to IIB CC, outcomes were overall survival (OS) and disease-free survival (DFS), and available or calculable hazard ratios (HRs) and 95% confidence intervals (CIs). Additionally, we excluded the following articles: letters, meeting summaries, commentary articles, posters, and those with unavailable results.

### 2.3. Data collection

Two authors collected data, and another author was invited to negotiate in case of any dispute. Data features included first author, publication year, subject origin, study design, case number, tumor stage, neoadjuvant therapy, threshold generation approach, cancer-specific results, LVSI threshold, and HRs with 95% CIs. This study mostly analyzed the performance of LVSI in the prognosis prediction in patients with CC.

### 2.4. Data handling and statistical analysis

We analyzed the association of LVSI with survival in patients with stage IA to IIB CC. Pooled HRs and 95% CIs were used to assess CC survival based on the same approach used in our previous study.^[15]^ DFS is defined as the time from the first treatment to the date of disease progression, suggesting the period after successful treatment with no symptoms or disease effect.^[16]^ In this meta-analysis, we pooled progression-free survival (PFS) and recurrence-free survival (RFS) from the enrolled articles into DFS. Moreover, OS is defined as the duration from the first treatment to all-cause mortality.^[16,17]^ We obtained multivariate HRs and 95% CIs and used univariate HRs to replace missing HRs from multivariate analysis. If HRs from uni- and multivariate analyses could not be obtained, the HRs were estimated using the approach of Parmar et al^[18]^ Kaplan–Meier method was used to analyze related variance using Engauge Digitizer (version 9.4). High LVSI plus HR of >1 and <1 were associated with dismal and good prognoses, respectively. Moreover, statistical heterogeneity was analyzed using chi-square *Q* test and *I*^2^ statistics. Obvious heterogeneity was determined by *P* < .05 and *I*^2^ > 50%, suggesting the adoption of a random-effects model; otherwise, the fixed-effects model must be used. RevMan version 5.3 (The Nordic Cochrane Centre, The Cochrane Collaboration) and STATA version 12.0 (STATA Corp., College Station, TX) were used for statistical analysis. Begg and Egger’s tests used STATA version 12.0 to evaluate bias. A *P* value of <.05 was considered statistically significant.

## 3. Results

### 3.1. Results of the study collection

**Figure 1.**
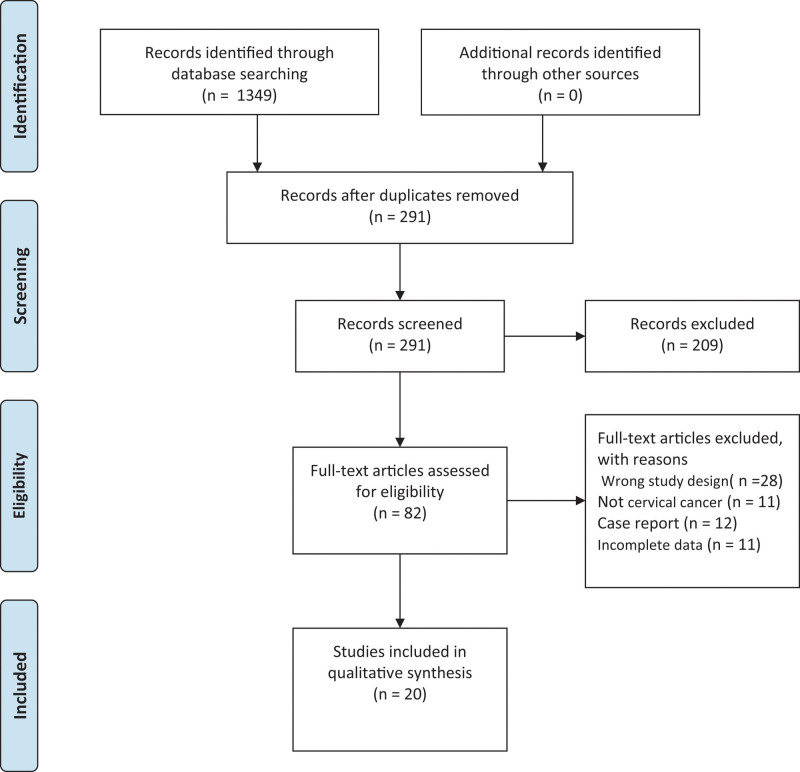
Flow diagram of study selection.

Databases, including Embase, PubMed, and Cochrane Library, were searched, obtaining 850, 499, and 0 articles, respectively. Thereafter, meeting summaries and duplicates were removed, leaving 82 qualified articles. Of the 82 articles, 62 were eliminated because of unwanted study design (n = 28), case reports (n = 12), non-relevance to CC (n = 11), and unavailable creditable data (n = 11). Finally, 20 qualified articles involving 25,352 cases in 2010 to 2021 were included in the meta-analysis^[11–13,19–35]^ (Fig. 1).

### 3.2. Study characteristics

All eligible articles published in 2010 to 2021 were retrospective, with a sample size of 47 to 10,314. Fifteen studies were from Asia (nine from China, one from Japan, and five from Korea), one from Belgium, one from France, and one from Italy. Each article analyzed stage IA to IIB CC cases, including treatments and histological characteristics. The follow-up duration was 3 to 338 months. Table 1 displays data on enrolled studies, such as treatment or histology.

**Table 1:** Included article characteristics.

Study	Year	Year of sample collection	Country	Study design	Sample size	Follow up period (years; median, IQR)	Tumour stage	Cutoff generating approach	Cancer-specific outcomes	Histologic type	Therapy
Jolien Haesen et al	2021	1997–2017	Belgium	R	182	13 (8–17) yr	IB1	Others	OS, PFS	Laparotomy Laparoscopy Robot-assisted	Surgery Radiotherapy Chemoradiotherapy
Chenyan Guo et al	2020	2006–2017	China	R	3986	90 (18–162) mo	IA1-IIA1	Others	OS, RFS	SCC AC AS Rare type	Surgery Radical hysterectomy Chemoradiation Therapy
Biliang Chen et al	2020	2004–2016	China	R	10,314	–	IA1-IIA2	Others	OS, DFS	SCC AC AS	Surgical Chemotherapy Radiotherapy/radiochemotherapy
Ju-Hyun Kim et al	2020	1993–2017	Korea	R	47	28.2 mo (3.83−202.5)	IB1-IIA1	Others	OS, DFS	Small Large	Surgery RT
Lijie Cao et al	2020	2006–2014	China	R	861	63 (45 84) mo	IB1-IIA2	Others	RFS	SCC	Radiotherapy Chemoradiotherapy
Benoit Bataille et al	2019	2000–2013	France	R	80	6.7 (5.4–8.5) yr	IB1-IIA	Others	DFS	SCC AC	Surgery RT
Lijie Cao* et al	2019	2006–2014	China	R	5181	59 (32–82) mo	IA2-IIA2	Others	OS, DFS	SCC AC AS	Surgery Chemotherapy Radiotherapy Chemoradiotherapy
Wan Kyu Eo et al	2018	2005–2013	Korea	R	233	46.6 (9–142) mo	IB-IIA	Others	OS, PFS	SCC AC AS	Surgery Radiotherapy chemotherapy
Antonino Ditto et al	2018	2001–2015	Italy	R	652	123 (1–338) mo	IA-IIB	Others	DFS	SCC AC Other type	Surgery Radiotherapy Chemotherapy
Bangxing Huang et al	2016	2008–2014	China	R	643	37 (11–97) mo	IA2-IIA	ROC	OS, DFS	SCC AC Others	Surgery Radiotherapy Chemotherapy
Miao-fang Wu et al	2016	2005–2010	china	R	839	36 mo	IB-IIA	Others	RFS	SCC Other	Surgery Radiotherapy Chemotherapy
Jing Li et al	2016	2005–2010	China	R	418	37.5(4–65) mo	IB-IIA	Others	RFS	SCC	Surgery Radiotherapy Chemotherapy
F. Martinelli et al	2015	1990–2011	Italy	R	275	–	IB-IIB	Others	OS	SCC AC Other	Surgery Radiotherapy Chemotherapy
Fraukje J.M. Pol et al	2015	2000–2012	Netherlands	R	210	57 (10–136) mo	IA2-IB1	Others	DFS	SCC AC AS	Surgery Radiotherapy Chemotherapy
Shanshan Yang et al	2015	2008–2010	China	R	264	68.5 (5–84) mo	I–II	Others	OS, DFS	SCC AC	Surgery Radiotherapy Chemotherapy
YOO-YOUNG LEE et al	2013	1997–2007	Korea	R	75	59.0 ± 28.0 mo	IB-IIA	Others	OS, DFS	SCC ASC AC	Surgery Radiotherapy Chemotherapy
K Matsuo et al	2013	1998–2008	Japan	R	540	5.0 (0.5–5.1) yr	IA2-IIB	Others	OS, DFS	SCC AC AS	Surgery Radiotherapy Chemotherapy
Lin Gong et al	2012	2008–2009	China	R	414	14 (range 4–27) mo	IB2-IIB	Others	OS	SCC AC AS Special	Surgery Radiotherapy Chemotherapy
Hyun Hoon Chung et al	2011	2003–2008	Korea	R	63	–	IB-IIA	ROC	DFS	SCC AC AS	Surgery Radiotherapy Chemotherapy
Hyun Hoon Chung* et al	2010	2003–2008	Korea	R	75	13 (3–58) mo	IB-IIA	ROC	PFS	SCC AC AS	Surgery Radiotherapy Chemotherapy

AC = adenocarcinoma, AS = adenosquamous carcinoma, DFS = disease-free survival, OS = overall survival, R = retrospective, ROC = receiver operating characteristic, RT = radical trachelectomy, SCC = squamous cell carcinoma.

### 3.3. Study quality evaluation

**Figure 2.**
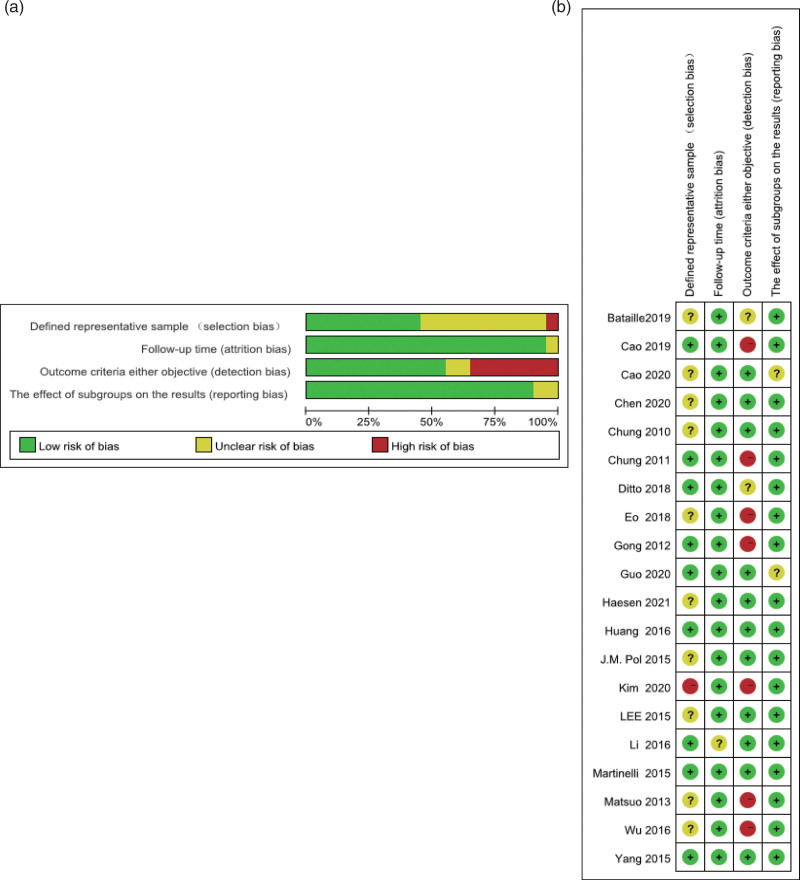
(A) Graph showing bias risk judgments on bias risk items through reviewers displayed percentage among all included studies. (B) Risk of bias summarization: risk of bias item judgment by reviewers for all included studies.

The study quality was assessed using Critical Appraisal of Prognostic Studies (https://www.cebm.net/wpcontent/uploads/2018/11/Prognosis.pdf; Fig. 2). Each article was assessed cautiously. The articles were retrospective with high quality. One article was a high risk; one had high bias risk; and ten had unknown bias risk due to non-blinded and non-randomized study design. Meanwhile, 1 study had an unknown bias risk, which was probably due to missing relapse or median follow-up information. Additionally, 7 studies were associated with a high bias risk, whereas two had unclear bias risk, which was possibly due to the outcome criteria or objective. Finally, most of our enrolled articles reported side effects objectively.

#### 3.3.1. Primary endpoint: DFS.

**Figure 3.**
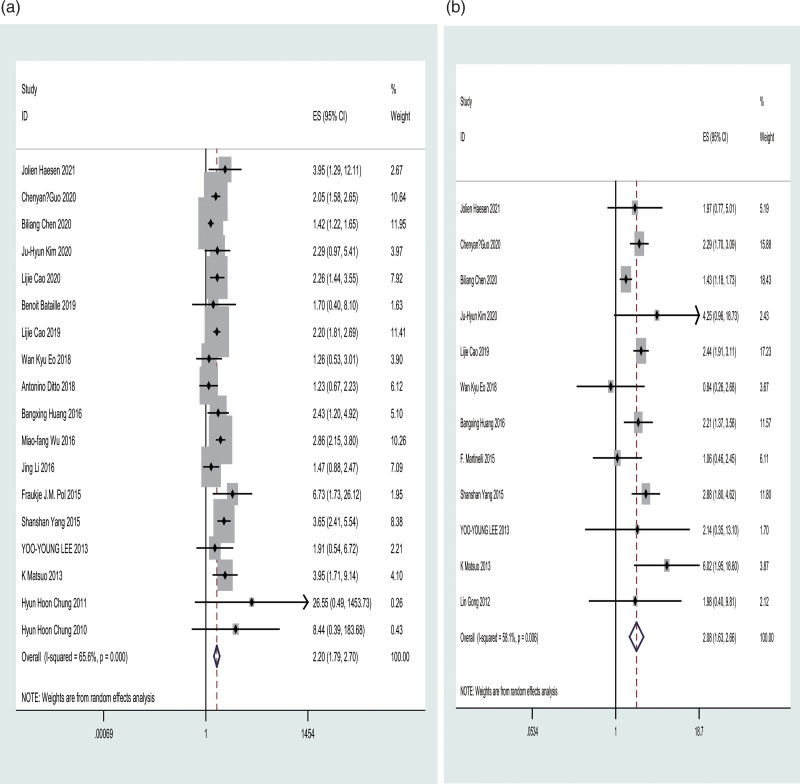
Forest plots showing HRs of DFS and OS as a function of LVSI (A, DFS; B, OS). The chi-square test was used to detect heterogeneity, where *P* < .05 indicated distinct heterogeneity between studies. Horizontal lines = 95% CI. (Fixed: fixed-effects model; Horizontal lines = 95% CI. Rhombus = estimates with corresponding 95% CI. Squares = individual study point estimates). DFS = disease-free survival, HRs = hazard ratios, LVSI = lymphovascular space invasion, OS = overall survival.

**Figure 4.**
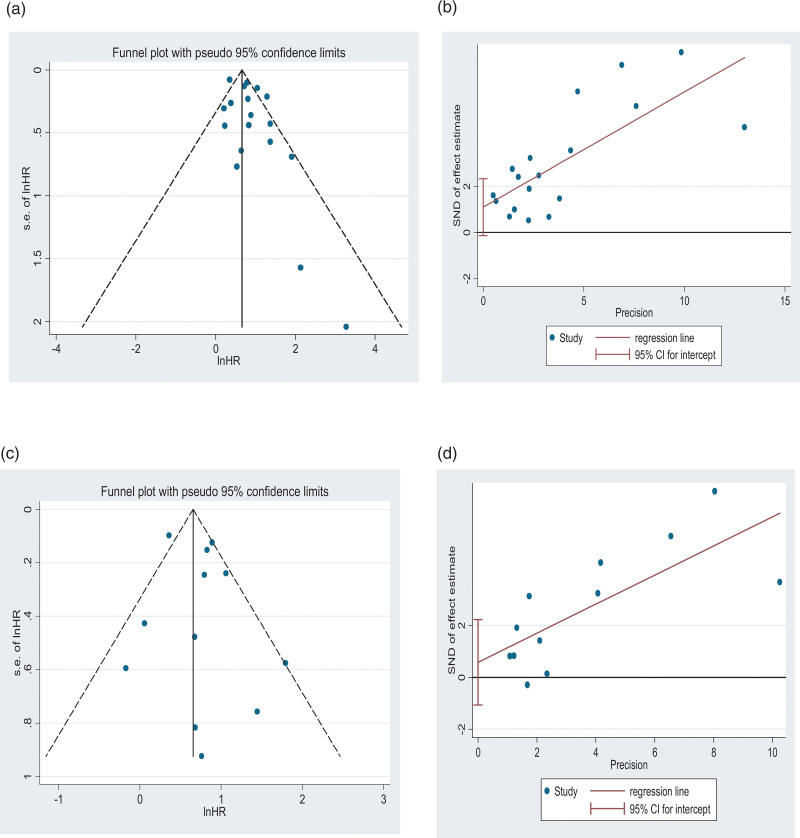
Funnel plots for EFS and OS with LVSI (A, DFS; C, OS) and Egger test for EFS and OS with LVSI (B, DFS; D, OS) The pseudo 95% CI was computed as part of the analysis to produce the funnel plots and correspond to the expected 95% CI for a given SE. Pseudo 95% CI was also determined to producefunnel plots and relevant 95% CI for the specific SE. CI = confidence interval, LVSI = lymphovascular space invasion, OS = overall survival, SE = standard error.

Eighteen studies evaluated the association between LVSI and DFS. When HRs were combined, high and higher LVSI indicated poor and worse DFS, respectively. The fixed-effects model was used to determine statistical significance (HR = 1.94; 95% CI = 1.77–2.12; *P* = .000; *I*^2^ = 65.6%). Inter-study heterogeneity was detected, and the random-effects model was used to obtain significant outcomes (HR = 2.20; 95% CI = 1.79–2.70) (Fig. 3A). Moreover, sensitivity analysis was performed to predict the impact of an individual study on pooled HRs. The results remained almost unchanged after eliminating 1 article (Figure S1A, Supplemental Digital Content, http://links.lww.com/MD/I810), suggesting significant results. Moreover, the funnel plots showed no obvious publication bias (Fig. 4A). Subsequently, Egger and Begg’s tests detected no significant publication bias (*P* = .82, *P* = .077) (Fig. 4B). Moreover, we conducted a subgroup analysis stratified by region, threshold methods, and endpoint (Table 2). Based on region-stratified subgroup analysis, the HR of 14 Asian articles was 2.20 (95% CI = 1.77–2.73; *P* = .000), and 4 European articles showed obvious associations (HR = 2.47; 95% CI = 1.07–5.66). In method-stratified analysis, the HR of the studies on uni- and multivariate regression was 2.21 (95% CI = 1.87–2.63, *P* = .129) and 2.33 (95% CI = 1.74–3.12, *P* = .000), respectively. Endpoint-stratified analysis showed that qualified studies were classified into DFS, PFS, and RFS groups, indicating pooled HRs of 2.27 (95% CI = 1.67–3.08; *P* = .000), 2.07 (95% CI = 1.06–4.05; *P* = .189), and 2.25 (95% CI = 1.90–2.66; *P* = .119), respectively.

**Table 2:** Subgroup analysis on OS and DFS of LVSI.

Endpoint	Factor	No. of studies	Heterogeneity test (*I*^2^, P)	Effect model	HR	95% CI of HR	Conclusion
DFS	Region						
	Asian	14	69.4, 0.000	Random	2.20	1.77, 2.73	Significant
	Europen	4	57.2, 0.072	Random	2.47	1.07, 5.66	Significant
	Analysis method						
	Univariate analysis	7	39.3, 0.129	Fixed	2.21	1.87, 2.63	Significant
	Multivariate analysis	11	72.5, 0.000	Random	2.33	1.74, 3.12	Significant
	Endpoint						
	DFS	11	72.0, 0.000	Random	2.27	1.67, 3.08	Significant
	PFS	3	40.0, 0.189	Fixed	2.07	1.06, 4.05	Significant
	RFS	4	48.7, 0.119	Fixed	2.25	1.90, 2.66	Significant
OS	Region						
	Asian	10	62.9, 0.004	Random	2.19	1.68, 2.86	Significant
	Europen	2	0.0, 0.335	Fixed	1.39	0.75, 2.60	Insignificant
	Analysis method						
	Univariate analysis	5	34.3, 0.193	Fixed	2.04	1.57, 2.64	Significant
	Multivariate analysis	7	69.9, 0.003	Random	2.26	1.62, 3.16	Significant

CI = confidence interval, DFS = disease-free survival, HR = hazard ratio, LVSI = lymphovascular space invasion, OS = overall survival, P = prospective, ROC = receiver operating characteristic.

#### 3.3.2. Primary outcome: OS.

Twelve articles examined the association between OS and LVSI. When HRs were pooled, high and higher LVSI indicated poor and worse OS, respectively. The fixed-effects model showed a significant difference (HR = 1.93; 95% CI = 1.71–2.17, *P* = .006; *I*^2^ = 58.1%), with inter-study heterogeneity. Moreover, the random-effects model showed significant results (HR = 2.08; 95% CI = 1.63–2.66) (Fig. 3B). We conducted a sensitivity analysis to analyze the impact of an individual study on pooled HRs. Thus, the results remained unchanged after removing each article (Figure S1B, Supplemental Digital Content, http://links.lww.com/MD/I810), suggesting significant results. The funnel plots showed no obvious publication bias (Fig. 4C). No publication bias was detected after performing Egger and Begg tests (*P* = .631; *P* = .453) (Fig. 4D). Furthermore, subgroup analysis stratified by region and threshold methods was performed (Table 2). The region-stratified subgroup analysis showed that the HR for 10 Asian articles was 2.19 (95% CI = 1.68–2.86; *P* = .004), and 2 European articles did not show any significant relationship (HR = 1.39; 95% CI = 0.75–2.60). In the method-stratified analysis, the HRs of studies on uni- and multivariate regression were 2.04 (95% CI = 1.57–2.64, *P* = .193) and 2.26 (95% CI = 1.62–3.16, *P* = .003), respectively.

## 4. Discussion

The status of the lymph node has a critical effect on CC. Some studies indicate that positive LNM has a negative impact on patient survival, regardless of the stage at diagnosis.^[36,37]^ To illustrate that nodal status is significant, nodal disease (radiological and pathological) is listed in the updated International Federation of Gynecology and Obstetrics 2018 classification system, and it markedly upstages uterine cancer and CC from stage I to stage III.^[38]^ Additionally, LVSI can be used to predict cancer prognosis of non-small cell lung carcinoma, rectal carcinoma, and oral squamous cell carcinomas based on the associated meta-analyses.^[39–41]^

The LVSI burden in CC remains unclear. Patients with CC can benefit if LVSI level predicts OS and DFS. LVSI represents the better approach to predict prognosis because it integrates data on locoregional metastasis burden and neck dissection type, thus combining the advantages of both parameters and overcoming their disadvantages.^[9]^ To the best of our knowledge, this meta-analysis was the first to analyze the significance of LVSI in CC prognosis prediction. This study included 20 qualified articles describing the associations between CC and LVSI. Although LVSI might be influenced by varied reasons, high LVSI indicated poor DFS (HR = 2.20; 95% CI = 1.79–2.70; *P* = .000; *I*^2^ = 65.6%) and OS (HR = 2.08; 95% CI = 1.63–2.66; *P* = .006; *I*^2^ = 58.1%) in stage IA to IIB CC based on the pooled analysis.

Significant heterogeneity was found for LVSI in predicting DFS (*P* = .000; *I*^2^ = 65.6%). Sensitivity analysis was performed to predict whether an individual article affected the pooled HRs, which showed unchanged results after removing every article, suggesting significant results. Funnel plots and Egger and Begg tests were performed to analyze possible publication bias, and no significant publication bias was obtained. However, the relationship between LVSI and survival was possibly impacted by some confounders. Consequently, subgroup analysis stratified by region, analysis method, and the endpoint was performed for EFS to investigate the heterogeneity source. In the method-stratified subgroup analysis, univariate analysis showed statistical significance, but no heterogeneity was detected. Among the 3 subgroups showing diverse survival end points, only two showed significant differences in PFS (*I*^2^ = 40.0, *P* = .189) and RFS (*I*^2^ = 48.7, *P* = .119), but no heterogeneity was detected. Therefore, the analysis method, HR source, and endpoint were the heterogeneity sources for DFS. Likewise, heterogeneity was detected for OS in LVSI prediction (*I*^2^ = 58.1%; *P* = .006). We conducted a sensitivity analysis to analyze the effect of 1 individual article on pooled HRs, and the results remained unchanged when eliminating any 1 article, suggesting significant results. Significant publication bias was not detected after performing funnel plots and Egger and Begg tests. Therefore, we performed subgroup analysis stratified by region and analysis method for OS to investigate the heterogeneity source. Based on the region-stratified analysis, the European group showed statistical significance, but no heterogeneity was detected. Based on the analysis method-stratified subgroup analysis, the univariate analysis group showed statistical significance, and no heterogeneity was detected. Therefore, the analysis method, HR source, and endpoint were the possible heterogeneity sources for OS.

The article quality should be considered, which is our study’s limitation. First, the Cochrane risk bias approach was used to assess the included articles, and we included high-quality articles. However, some had incomplete patient data. Moreover, these articles were retrospective; thus, more prospective studies that combine CC survival with LVSI are warranted. Second, because of CC heterogeneity, patients with different histological grades and stages and those receiving diverse treatments were included, which might affect patient survival and event occurrence. Third, only articles published in the English language were included, which might induce language bias. Finally, published articles were included in database searching, which could lead to publication bias. However, our results were reliable upon publication bias analysis.

## 5. Conclusion

Patients with CC of different subtypes were administered with different treatments. Our study suggests that high LVSI predicts poor prognostic outcomes in patients with stage IA to IIB CC. However, our results should be further verified in larger, high-quality studies.

## Author contributions

**Conceptualization:** Yuan Huang, Xiangdan Li.

Data curation: Xiangdan Li.

Formal analysis: Yuan Huang, Xiangdan Li.

Funding acquisition: Xiangdan Li, Lan Liu.

Investigation: Weibo Wen, Xiangdan Li, Dongyuan Xu, Lan Liu.

Methodology: Yuan Huang, Weibo Wen, Xiangdan Li, Dongyuan Xu, Lan Liu.

Project administration: Yuan Huang, Weibo Wen, Dongyuan Xu, Lan Liu.

Resources: Weibo Wen, Dongyuan Xu, Lan Liu.

Software: Weibo Wen, Dongyuan Xu, Lan Liu.

Supervision: Weibo Wen, Dongyuan Xu.

Validation: Weibo Wen, Dongyuan Xu.

## Supplementary Material


