# Supplementary material for: Prognostic value of lymphovascular space invasion in stage IA to IIB cervical cancer: A meta-analysis
Source: Medicine (Baltimore). 2023 Apr 14;102(15):e33547. doi: 10.1097/MD.0000000000033547 (PMC10101290; doi:10.1097/MD.0000000000033547)

Supplementary Figure 1.Sensitivity analysis of DFS and OS with LVSI (A, DFS; B, OS).

LVSI = lymphovascular space invasion,DFS = disease-free survival,OS = overall survival.

A

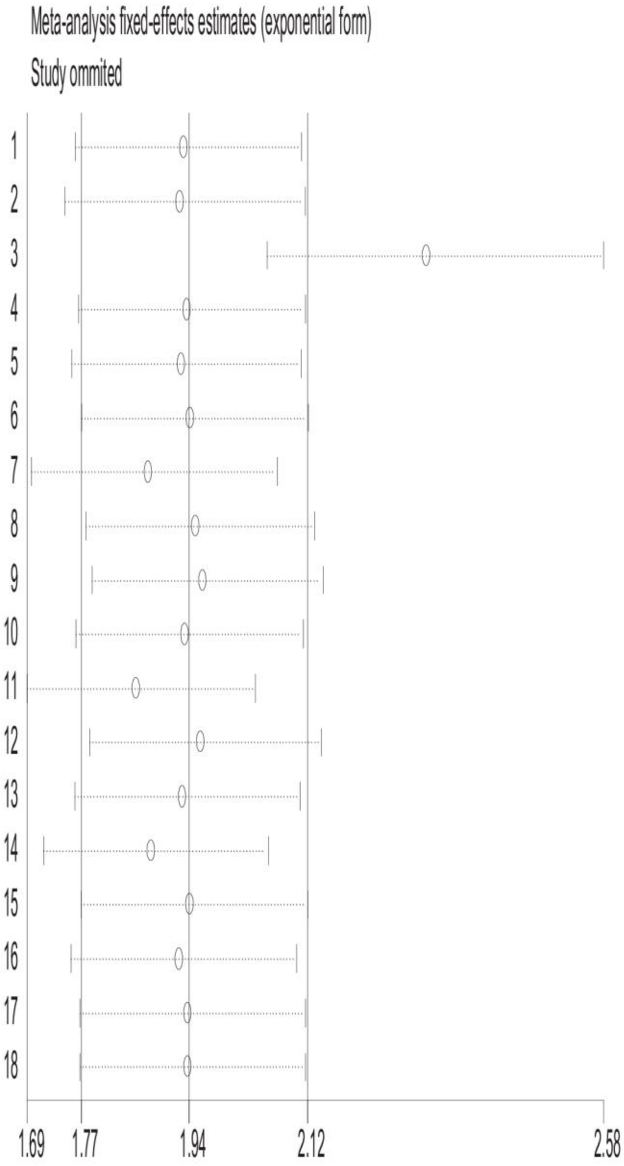

B

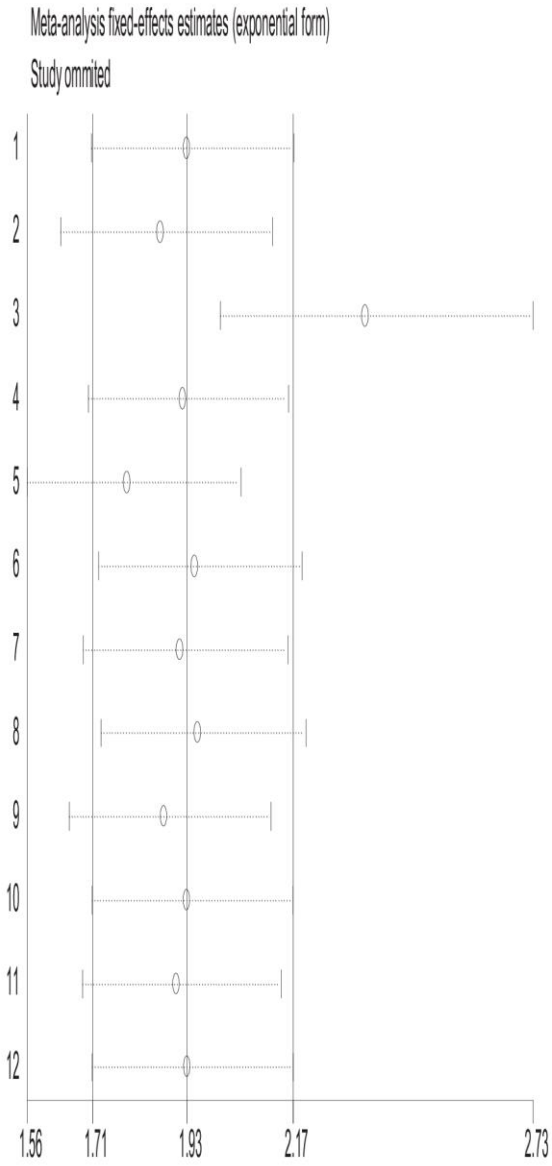

Supplement: Supplementary file 1 [file medi-102-e33547-s001.pdf]
